# Diagnostic accuracy of T2-hypointensity in determining the epileptogenic lesion on unmyelinated brain MRI in infants with tuberous sclerosis complex (TSC)

**DOI:** 10.1093/braincomms/fcaf241

**Published:** 2025-06-26

**Authors:** Matyas Ebel, Carmen H Stevering, Zuzana Holubova, Maarten H Lequin, Martin Kyncl, Barbora Straka, Hanna M Hulshof, Alena Jahodova, Martin Kudr, Kees P J Braun, Floor E Jansen, Pavel Krsek

**Affiliations:** Department of Paediatric Neurology, Motol Epilepsy Center, Second Faculty of Medicine, Charles University and Motol University Hospital, Prague 150 06, Czech Republic; Department of Paediatric Neurology, University Medical Center Utrecht Brain Center, PO Box 85090 AB 3508, Utrecht, The Netherlands; Department of Radiology, Second Faculty of Medicine, Charles University and Motol University Hospital, Prague 150 06, Czech Republic; Department of Radiology, University Medical Center Utrecht, Utrecht CX 3584, The Netherlands; Department of Radiology, Second Faculty of Medicine, Charles University and Motol University Hospital, Prague 150 06, Czech Republic; Department of Radiology, Second Faculty of Medicine, Charles University and Motol University Hospital, Prague 150 06, Czech Republic; Department of Paediatric Neurology, University Medical Center Utrecht Brain Center, PO Box 85090 AB 3508, Utrecht, The Netherlands; Department of Paediatric Neurology, Motol Epilepsy Center, Second Faculty of Medicine, Charles University and Motol University Hospital, Prague 150 06, Czech Republic; Department of Paediatric Neurology, Motol Epilepsy Center, Second Faculty of Medicine, Charles University and Motol University Hospital, Prague 150 06, Czech Republic; Department of Paediatric Neurology, University Medical Center Utrecht Brain Center, PO Box 85090 AB 3508, Utrecht, The Netherlands; Department of Paediatric Neurology, University Medical Center Utrecht Brain Center, PO Box 85090 AB 3508, Utrecht, The Netherlands; Department of Paediatric Neurology, Motol Epilepsy Center, Second Faculty of Medicine, Charles University and Motol University Hospital, Prague 150 06, Czech Republic

**Keywords:** epilepsy surgery, TSC, myelination, MRI

## Abstract

Identification of the epileptogenic lesion is challenging in tuberous sclerosis complex as multiple lesions might represent the seizure onset zone. A combination of dysplastic MRI features has diagnostic value in pre-surgical evaluation. However, these radiological characteristics may be difficult to identify and have not been studied on early unmyelinated brain MRI in tuberous sclerosis complex infants. Our study aimed to assess the diagnostic accuracy of T2-hypointense lesions on unmyelinated MRI in identifying the epileptogenic lesion. We included children with tuberous sclerosis complex who underwent resective or disconnective epilepsy surgery in the Motol University Hospital Prague and the University Medical Center Utrecht with available (i) unmyelinated MRI (before the age of 9 months), (ii) pre- and post-operative brain MRI and (iii) at least 2 years follow-up post-surgery. We identified T2-hypointense lesions and highly dysplastic lesions on unmyelinated or myelinated MRI, assessing their diagnostic accuracy in epileptogenic lesion identification by comparing seizure free to non-seizure free patients. Twenty-seven patients met inclusion criteria. We identified 54 T2-hypointense lesions in 24 patients, 30 were already highly dysplastic on unmyelinated MRI, showing cortical thickening and transmantle sign in most cases, while calcifications appeared later. Diagnostic accuracy of T2-hypointense (70.8%) was superior to the presence of the most dysplastic features (55.6%) in epileptogenic lesion identification. Positive predictive value for complete resection of all T2-hypointense lesions was 63.6%, compared to 50.0% for highly dysplastic lesions. Seizure recurrence was high (negative predictive value 76.9%) when T2-hypointense lesions remained outside the resected area. Assessing T2-hypointense lesions on unmyelinated brain MRI has important diagnostic value in identifying the epileptogenic lesion in pre-surgical work-up in infants with tuberous sclerosis complex and drug-resistant epilepsy. Unmyelinated brain MRI deserves a more important position in pre-surgical evaluation in infants with tuberous sclerosis complex and drug-resistant epilepsy.

## Teaser

Ebel and Stevering *et al.* report that T2-hypointensity on unmyelinated brain MRI has significant diagnostic value in identifying epileptogenic lesions in infants with tuberous sclerosis complex (TSC). The study highlights the importance of early MRI evaluation in the pre-surgical work-up of infants with TSC and drug-resistant epilepsy.

## Graphical Abstract

**Graphical Abstract:**
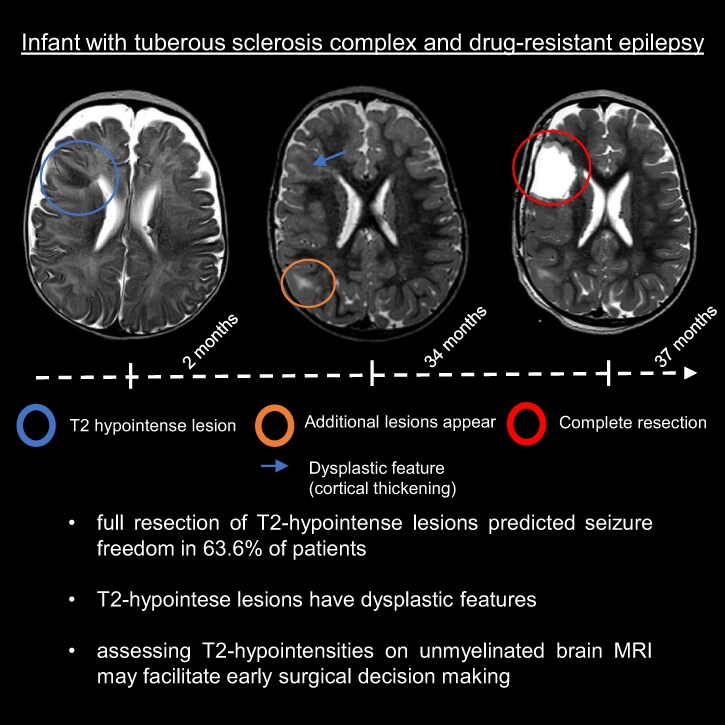

## Introduction

Tuberous sclerosis complex (TSC) is a multi-system disorder caused by pathogenic variants in *TSC1* or *TSC2* genes.^1^ The hallmark of brain MRIs in patients with TSC is the presence of tubers—a subtype of focal cortical dysplasias (FCD)—which cause a number of neurological and cognitive symptoms, including epilepsy in 85% of patients.^1^ Epilepsy is diagnosed in around 80% of patients before the age of two^2^ and is drug-resistant in 62%^1^ of the TSC patients. These patients may significantly benefit from epilepsy surgery,^1,3^ which results in seizure freedom in 55–65% of patients.^4-6^

Identification of the epileptogenic lesion (EL) in TSC poses a challenge during pre-surgical evaluation and surgical planning, due to the widespread localization of the numerous lesions. Shorter duration of epilepsy is associated with better outcomes in terms of seizure freedom and development,^7^ and epilepsy surgery is thus to be considered especially in younger TSC patients with refractory epilepsy who are at highest risk of developmental sequelae,^8^ and have a high burden of recurrent seizures and polypharmacy. Pre-surgical evaluation combines multiple imaging^9-11^ and electrophysiological^12^ modalities to approximate the delineation of the epileptogenic zone.

We previously demonstrated the importance of a detailed evaluation of MRI findings for the correct localization of the EL in TSC, as a combination of features consistent with FCD—including increased cortical thickness, grey-white matter blurring, transmantle sign and calcifications—improves the prediction of the localization of the EL.^11,13^

In infants with TSC the specific diagnostic challenges are even higher since the MRI evaluation of TSC-associated lesions and their dysplastic features on unmyelinated brain tissue is particularly demanding. Moreover, scalp EEG is often not localizing, or shows multi-focal epileptiform activity, and stereo EEG is very challenging before the age of 2 years.^14,15^ Early and precise definition of the EL in TSC infants would facilitate surgical decision making, enabling earlier (post-operative) seizure freedom and improvement of developmental outcome.

**Figure 1:**
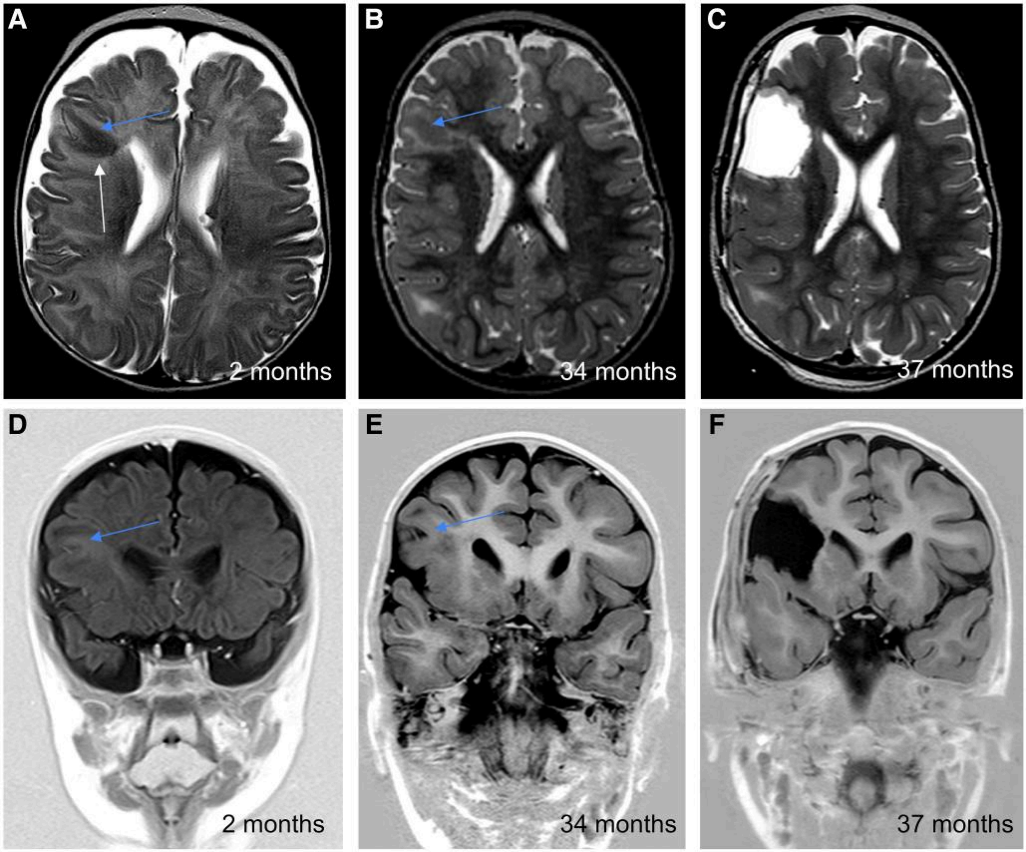
**Example of evolution of a T2HI lesion on unmyelinated brain MRI to a highly dysplastic lesion on myelinated brain MRI.** These MRI images show the evolution of T2-hypointensity (white arrow) in the right fronto-opercular region. Axial T2-weighted images at 2 months (**A**, unmyelinated) and 34 months (**B**, myelinated), with corresponding coronal T1-weighted images (**D** and **E**). Note the persistent cortical thickening (blue arrows). Post-surgical images (**C**, axial T2; F, coronal T1) at 37 months confirm complete lesion resection. The patient has remained seizure-free since surgery. The patient is seizure-free since surgery.

A report by Eltze *et al*.^16^ showed that the MRI signal of FCD type II lesions changes with ongoing myelination. Given that TSC-associated brain lesions often harbour multiple FCD type II-like imaging features,^11,13^ we hypothesized that with ongoing myelination brain lesions in TSC may undergo similar changes to FCD type II and therefore T2-hypointense (T2HI) tubers (Fig. 1) are likely epileptogenic,^16^ which was supported by our clinical experience. The aim of this study was to evaluate the diagnostic value of a lesion being T2HI on unmyelinated brain MRI in defining the EL in TSC patients undergoing epilepsy surgery.

## Materials and methods

### Study population and data acquisition

In this retrospective consecutive observational study, patients were included from the Motol University Hospital, Prague, Czech Republic (MUH) and the University Medical Center Utrecht, the Netherlands (UMCU). Patients with a definite clinical or a genetic diagnosis of TSC^17^ who underwent resective or disconnective epilepsy surgery between 2006 and 2020, were included if they fulfilled the following criteria: (i) availability of good-quality pre-surgical MRI with below-specified technical requirements, performed before the age of 9 months; (ii) availability of at least one pre- and post-operative MRI; (iii) clinical follow-up data of at least 2 years post-surgery. There were no additional exclusion criteria applied.

MRI scans were required to be performed on at least 1.5T field strength MRI scanner, with T1W, T2W and FLAIR images acquired in at least two different planes with a maximum slice thickness of 5 mm (2 mm or 3D images were available in most cases). Clinical characteristics including TSC variant, age at clinical seizure onset, age at epilepsy surgery, details about surgical procedures and seizure outcomes according to Engel classification were available in all patients. In cases with post-operative seizure recurrence, we determined the likely reason for surgical failure, as judged by the clinical epilepsy surgery team based on post-operative MRI, seizure semiology, and EEG results.

Surgical planning was based on multi-modal data evaluation (seizure semiology, neurological status, electrophysiological, and neuroimaging findings). Delineation of the EL was never solely based on MRI findings. The study was approved by medical ethic committees at both MUH and UMCU, which concluded that the Medical Research Involving Humans Act (WMO) did not apply and informed written consent was not required.

### Study design

To study the evolution of dysplastic lesions on brain MRI, we evaluated three different categories of MRIs depending on brain myelination and time course: (i) pre-surgical MRI of so-called ‘unmyelinated’ brain, (ii) pre-surgical MRI of ‘myelinated’ brain (when available) and (iii) post-surgical MRI (myelinated or unmyelinated, depending on a patient’s age at surgery). Unmyelinated brain MRI was defined as performed before the age of 9 months, as peripheral white matter becomes myelinated as visualized on imaging after the age of 9 months.^18^ Myelinated brain MRI was defined as performed at the age of 1 year and older (in the majority after the age of 2 years). While myelinated brain pre-surgical MRI was not mandatory, it enabled us to assess the evolution of some of the resected lesions. We excluded the MRIs from the transitional period between 9 and 12 months from the evaluation due to poor differentiation between white and grey matter during this time.^19^ Although myelination is not entirely complete by this age, our observations indicate that most lesions significantly change their appearance within that interval.

**Figure 2:**
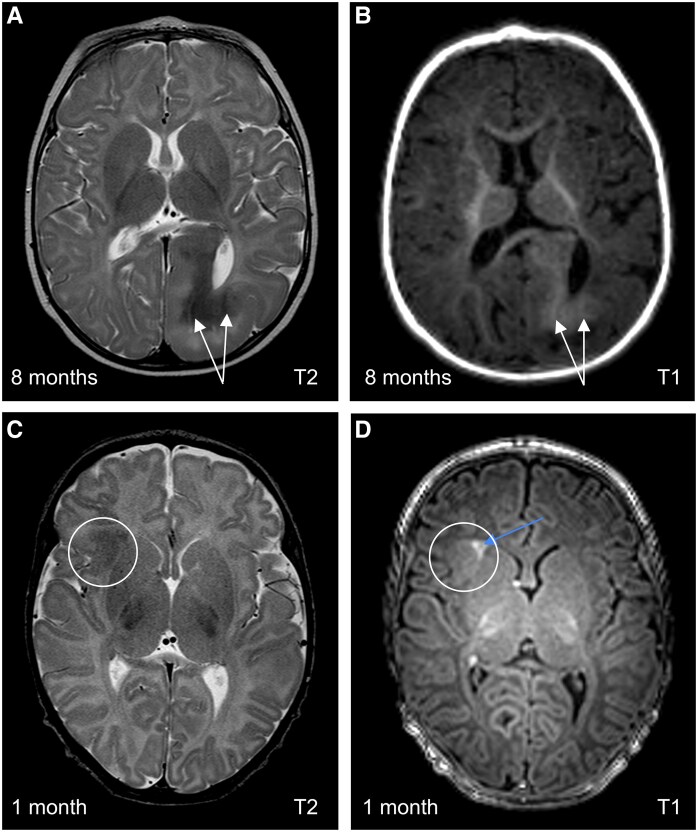
**Calcified tuber on unmyelinated MRI.** Comparison of the calcified and uncalcified T2-hypointense lesion on axial scans. Patient 23. Calcified lesion in the left occipital lobe. On T2-weighted image **(A)** calcified areas are noticeably hypointense irregularities compared to surrounding T2-hypointense lesion, hyperintense on T1-weighted image, respectively (**B**) (white arrows). Patient 27. Lesion in the right frontal lobe is without calcification (circle). T2-weighted image **(C)** displays homogeneously hypointense lesion, slightly hyperintense on T1-weighted image **(D)**. Strikingly T1 hyperintense transmantle sign (blue arrow) can be seen as well.

MRIs were evaluated by a team of experts in both participating centres independently (MK and ZH in MUH; CS, ML and FJ in UMCU), as our previous study provided evidence of a good inter-observer agreement between neuroradiologists from the two epilepsy centres.^13^ On unmyelinated MRI we assessed all T2HI lesions and all lesions that were at first or later MRI considered ‘highly dysplastic’, defined by having at least two of the following features: cortical thickening, transmantle sign, calcifications, or a lesion being the largest. Our previous study revealed a combination of two or more of these features as being predictive for epileptogenicity of the tuber.^11,13^ Cystic lesions were not evaluated due to their inconsistent association with EZ^13^ and the poor inter-observer reliability of their assessment.^11^ Calcifications, also characterized by decreased T2 signal intensity, were distinguished from T2HI lesions in our study by their distinct imaging features, as illustrated in the Fig. 2, however subtle calcifications on the unmyelinated MRI may not be reliably identified without dedicated sequences. The largest lesion was identified visually. On pre-surgical MRI, we evaluated dysplastic features of the lesions previously identified on unmyelinated pre-surgical MRI and additionally assessed lesions that were not previously detected but met definition of being highly dysplastic according to the criteria mentioned above with myelination. On post-surgical MRI, we evaluated the same descriptors in all previously marked lesions and analysed whether the area defined as the EL during pre-surgical decision-making and planning was completely resected or disconnected based on radiological imaging. For the purpose of the present study, we considered the lesion to be the EL if it was completely included in the resected area in patients who achieved post-operative seizure-freedom after 2 years. The evaluation was conducted chronologically, starting with the unmyelinated MRI and followed by the myelinated MRIs. Lesions that were considered highly dysplastic on myelinated MRI were reassessed on the unmyelinated MRI for their dysplastic features.

### Study endpoints

The main aim of this study was to determine the diagnostic value of a lesion being T2HI on unmyelinated MRI in defining the EL. Positive predictive value (PPV), negative predictive value (NPV) and diagnostic accuracy (defined as sensitivity × prevalence + specificity × (1-prevalence) were considered most clinically relevant, but other diagnostic values (sensitivity, specificity) were also evaluated. We performed the following analyses to identify the diagnostic accuracy of T2HI lesions in localizing the EL:

1. Predictive value of T2-hypointensity on early ‘unmyelinated’ MRI in defining the EL; i.e. all T2HI lesions were completely in the resected or disconnected area in post-operative seizure-free patients.
2. Predictive value of highly dysplastic lesion(s), defined as the presence of ≥ 2 or more dysplastic features in a lesion on ‘unmyelinated’ or ‘myelinated’ MRIs; i.e. the lesion with the most dysplastic features was completely in the resected or disconnected area in post-operative seizure-free patients. In case of equal number of dysplastic features in two or more lesions, the largest lesion was selected.

### Statistical analysis

Statistical analysis was performed using Matlab R2021b with Statistic and Machine learning Toolbox. Baseline characteristics were presented using descriptive statistics. Diagnostic accuracy of use of T2-hypointense lesions in identifying the EL was calculated using contingency tables, which allowed for the calculation of sensitivity, specificity, PPV and NPV. Diagnostic metrics are presented with 95% confidence intervals. For categorical variables association, Fisher’s exact test with the significance threshold *P* < 0.05 was applied.

## Results

### Clinical cohort

Sixty operated TSC patients (38 from UMCU and 22 from MUH) were screened for inclusion. Twenty-seven patients (16 from UMCU and 11 from MUH) met the inclusion criteria for the study, of whom 74,1% had a *TSC2* variant. Baseline characteristics of the study participants are presented in Table 1. The selected patient cohort only partially overlapped with our previous study, due to different inclusion criteria. The median of age at clinical seizure onset and age at surgery were 2.0 (0.1–3.5) and 23.0 (10.0–40.0) months, respectively. Twelve patients were seizure-free at the 2-year follow-up (44%), of whom five had completely withdrawn anti-seizure medication by then. Significant seizure reduction was achieved in 12 patients with Engel outcome score class II or III. Surgical failure was caused by relapse from another EL in 33.3% of cases, while in 60% the supposed reason for seizure recurrence was a larger extent of the EL. Seizure outcome was not dependent on the TSC mutation type (Fischer’s exact test, *P* = 1.00).

**Table 1:** Baseline characteristics of all included patients; in total and per seizure outcome according to the Engel classification

	Total (*n* = 27)	Post-operative seizure outcome—Engel classification			
		I (*n* = 12)	II (*n* = 2)	III (*n* = 10)	IV (*n* = 3)
TSC mutation type^a^					
*TSC1* (pathogenic)	6/27 (22.2%)	3/6 (50.0%)	1/6 (16.7%)	2/6 (33.3%)	0
*TSC2* (pathogenic)	20/27 (74.1%)	8/20 (40.0%)	1/20 (5.0%)	8/20 (40.0%)	3/20 (15.0%)
Unknown	1/27 (3.7%)	1 (100%)	0	0	0
Gender male/female	17/10				
Age at seizure onset (months)^b^	2.0 (0.1–3.5)	1.2 (0.8–2.3)	1.5 (0.8–2.3)	1.3 (0.3–2.0)	3.0 (3.0–3.5)
Age at unmyelinated MRI (months)^b^	2.0 (1–5)	3.0 (1.8–5.5)	1.5 (0.8–2.3)	1.5 (0.3–5.0)	2.0 (2.0–3.0)
Age at myelinated pre-surgical MRI (months)^b^	29.0 (23.0–57.0)	37 (21.5–48.0), NA 5/12	NA 2/2	27.0 (22.5–45.5), NA 3/10	32.0 (33–44.5)
Number of T2HI lesions^a^					
None	3/27 (11.1%)	2 (66.7%)	0 (0.0%)	0 (33.3%)	1 (11.1%)
Solitary	10/27 (37.0%)	7/10 (70.0%)	1/10 (10.0%)	2/10 (20.0%)	0 (0.0%)
Multiple	14/27 (51.9%)	3 (21.4%)	1 (7.1%)	8 (57.1%)	2 (14.3%)
Age at surgery (months)^b^	23.0 (10.0–40.0)	20.0 (8.5–39.0)	4.0 (3.0–5.0)	24.5 (17.3–45.3)	32.0 (30.5–48.5)
Age at post-surgical MRI (months)^b^	32.0 (20.5–56.5)	25.0 (11.3–43.5)	12.0 (9.5–14.5)	34.5 (29.8–64.3)	56.0 (50.5–75.0)
Surgery type^a^					
Focal resection	12/27 (44.4)	6/12 (50.0%)	1/12 (8.3%)	5/12 (41.7%)	0
Hemispherectomy	4/27 (14.8)	1/4 (25.0%)	1/4 (25.0%)	2/4 (50.0%)	0
Lobectomy	6/27 (22.2)	2/6 (33.3%)	0	1/6 (16.7%)	3/6 (50.0%)
Multi-lobar	5/27 (18.5)	3/5 (60.0%)	0	2/5 (40.0%)	0
Reason for surgical failure^a^					
Incomplete resection	9/15 (60.0%)	0	0	6/9 (66.7%)	3 (33.3%)
Relapse from another EL	5/15 (33.3%)	0	2/5 (40.0%)	3/5 (60.0%)	0
Unknown	1/15 (6.7%)	0	0	1 (100.0)	0

*n*, number of patients; TSC, tuberous sclerosis complex; MRI, magnetic resonance imaging; T2HI, T2 hypointense; EL, epileptogenic lesion.

^a^Data represented as number of patients (percentage).

^b^Data represented as median (inter-quartile range).

Characteristics which were regarded as outcome-independent were omitted to enhance clarity.

### Dysplastic features and evolution of T2HI lesions

**Figure 3:**
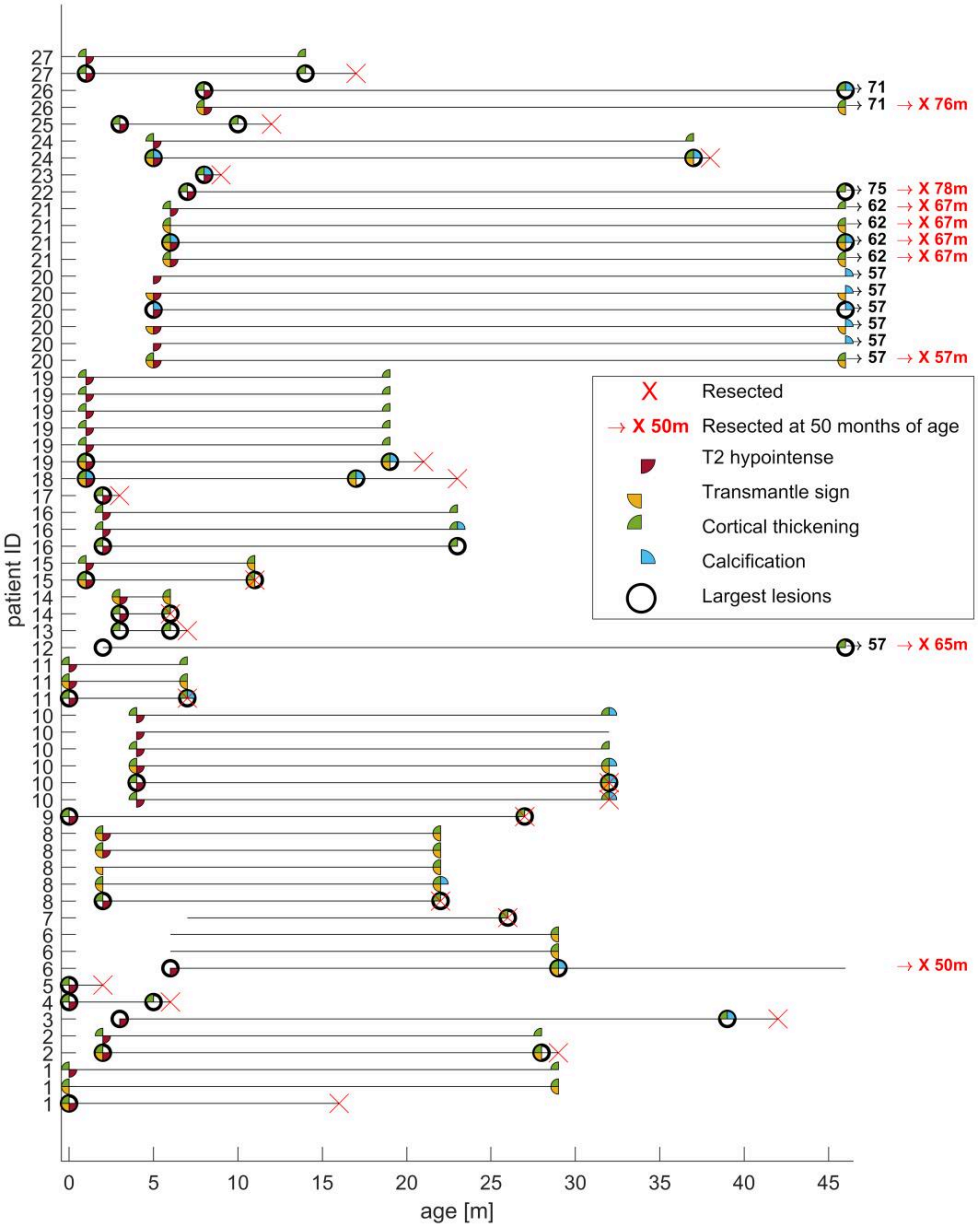
**Evolution of included MRI lesions during myelination until last pre-operative brain MRI legend.** This figure documents the evolution of all evaluated MRI lesions included in this study.

We identified 54 T2HI lesions on unmyelinated MRI in 24 of the total 27 patients; which were solitary in 10/24 patients and multiple in 14/24. Co-occurrence of other dysplastic features in each of these lesions on unmyelinated brain MRI and evolution of dysplastic features after myelination are presented in Fig. 3. Figure 3 shows all lesions that were either T2HI at first MRI, or had 2 or more dysplastic features at any time. Of the 54 T2HI lesions on unmyelinated MRI 30 lesions were already considered highly dysplastic on unmyelinated MRI, and 8 lesions appeared visible as highly dysplastic at later imaging, with ongoing myelination. Evolution of nine T2HI lesions in nine patients could not be assessed because of unavailability of pre-operative myelinated MRI, those all had cortical thickening, one was also calcified and two with transmantle sign. No T2HI lesion observed on the initial MRI remained T2HI on the latest MRI and no dysplastic features apparent on unmyelinated pre-surgical MRI disappeared throughout the myelination. There were four lesions on unmyelinated MRI that were not T2HI, but presented already with two or more dysplastic features.

Out of the 53 lesions evaluated initially (45 T2HI and 8 non-T2HI) and on the myelinated MRI, cortical thickening was present in 40 lesions on unmyelinated MRI and in 47 on myelinated MRI; the transmantle sign was seen in 19 lesions on unmyelinated MRI, and 24 lesions after myelination; and calcification was initially present in 4 lesions, and in 18 later. This suggests that cortical thickening and transmantle sign presented already on unmyelinated MRI in most cases, while calcification typically appeared on myelinated MRI, and only in a minority of lesions. There were three lesions that were not identified on the unmyelinated MRI, but were highly dysplastic on the last examination. Lesions with only one dysplastic feature on the final MRI were not analysed in this study.

Evolution of a T2HI lesion with visualization of dysplastic features in one of our cases is shown as an example in Fig. 1.

### Diagnostic accuracy of a lesion being T2HI in predicting the EL localization

Diagnostic accuracy of a lesion being T2HI in determining the EL is presented in Table 2. Resection of all T2HI lesions was associated with post-operative seizure freedom (=PPV) in 63.6% (95% CI 41.0–81.5) of patients, whereas incomplete resection or resection of not all T2HI lesions resulted in seizure recurrence (=NPV) in 76.9% (95% CI 55.0–90.1). Diagnostic accuracy of T2-hypointensity in defining the EL was 70.8% (95% CI 48.9–87.4). In this cohort PPV for the highly dysplastic lesion with most dysplastic features in predicting the EL was 50.0% (95% CI 41.4–58.7, Table 2), NPV 80.0% (95% CI 33.9–96.9) and diagnostic accuracy 55.6% (95% CI 35.3–74.5). It is worth noting that specificity of T2-hypointensity was considerably higher than specificity of highly dysplastic features, 71.4% (95% CI 41.9–91.6) and 26.7 (95% CI 7.8–55.1) respectively, suggesting that chances for post-operative seizure freedom are better after resecting all T2HI lesions compared to resecting the lesion with most dysplastic features.

**Table 2:** Diagnostic accuracy of T2HI lesions, highly dysplastic lesion(s) and combination of T2-hypointensity and dysplastic features in defining the EL

Analysis 1. diagnostic accuracy of T2-hypointense lesion(s)		
	Seizure freedom + (*n* = 10)	Seizure freedom − (*n* = 14)
All T2HI lesions in resected area (*n* = 11)	7	4
(remaining) T2HI lesions (partially) outside resected area (*n* = 13)	3	10
Patients without T2HI lesions were excluded from the analysis (*n* = 3).		
$\begin{array}{cc} \begin{array}{c} \mathrm{diagnostic}\;\mathrm{accuracy}\\ 70.8\text{\%}(95\text{\%}\mathrm{CI}\;48.9\;\text{–}87.4) \end{array} & \{\begin{array}{cc} \mathrm{PPV}=63.6\text{\%}(95\text{\%}\mathrm{CI}\;41.0\;\text{–}81.5) & \mathrm{sensitivity}=70.0\text{\%}(95\text{\%}\mathrm{CI}\;34.8\;\text{–}93.3)\\ \mathrm{NPV}=76.9\text{\%}(95\text{\%}\mathrm{CI}\;55.0\;\text{–}90.1) & \mathrm{specificity}=71.4\text{\%}(95\text{\%}\mathrm{CI}\;41.9\;\text{–}91.6) \end{array} \end{array}$		

Analysis 2. diagnostic accuracy of the highly dysplastic lesion with most dysplastic features		
	Seizure freedom + (*n* = 12)	Seizure freedom − (*n* = 15)
Highly dysplastic lesion(s) with most dysplastic features on (un)myelinated MRI in resected area and in case of equal number the largest lesion (*n* = 22)	11	11
Highly dysplastic lesion(s) with most dysplastic features and in case of equal number the largest lesion on (un)myelinated MRI (partially) outside resected area (*n* = 5)	1	4
Patients without highly dysplastic lesions (≥2 dysplastic features), on unmyelinated or myelinated MRI, were excluded from this analysis (*n* = 0). A lesion being a tuber or T2HI were not included as dysplastic features themselves.		
$\begin{array}{cc} \begin{array}{c} \mathrm{diagnostic}\;\mathrm{accuracy}\\ 55.6\text{\%}(95\text{\%}\mathrm{CI}\;35.3\;\text{–}74.5) \end{array} & \{\begin{array}{cc} \mathrm{PPV}=50.0\text{\%}(95\text{\%}\mathrm{CI}\;41.4\;\text{–}58.7) & \mathrm{sensitivity}=91.7\text{\%}(95\text{\%}\mathrm{CI}\;61.5\;\text{–}99.8)\\ \mathrm{NPV}=80.0\text{\%}(95\text{\%}\mathrm{CI}\;33.9\;\text{–}96.9) & \mathrm{specificity}=26.7\text{\%}(95\text{\%}\mathrm{CI}\;7.8\;\text{–}55.1) \end{array} \end{array}$		

## Discussion

Assessment of T2-hypointensity on unmyelinated brain MRI had higher diagnostic accuracy in defining the EL than predicting epileptogenicity based on the presence of the lesion’s dysplastic features. Resection of all T2HI lesions resulted in post-operative seizure freedom in the majority of patients. T2-hypointensity can already be identified at an early age, on unmyelinated MRI, and is also visually determined more easily than each different dysplastic feature, in particular to the untrained eye, underlining the diagnostic applicability of T2-hypointensity.

**Figure 4:**
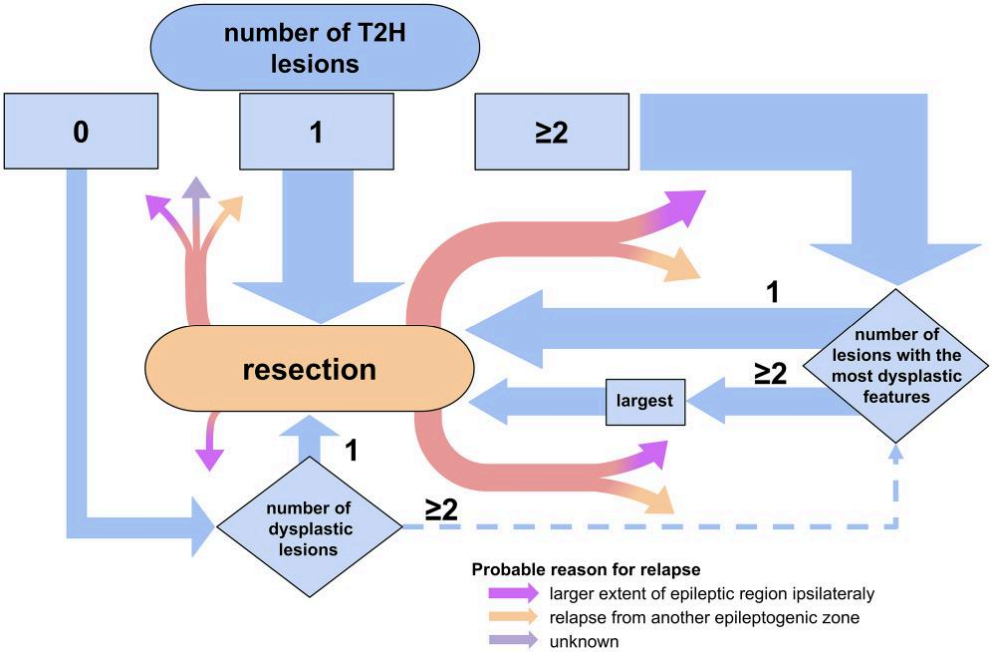
**Observed patterns of resection area selection: role of T2-hypointensity and dysplastic features.** This figure represents the usual trajectory of the patients in determining the location of resection. T2-hypointensity (T2HI) was the starting point for lesion selection, and information about additional dysplastic features was added in case of multiple T2HI lesions. In the case of the absence of or multitude of T2HI lesions, lesions the lesion with most dysplastic features was selected and in case of equal number of dysplastic features the largest lesion was chosen. The thickness of the arrows represents the number of patients proportionally. Three patients are not covered in the model (multiple T2Hl, resected one of those with the most dysplastic lesion, but not the largest, two of them are seizure-free).

Furthermore, combined information about T2-hypointensity and highly dysplastic features retrospectively corresponded to the resected area in most cases (Fig. 4). Specifically, solitary T2Hl were always included in the resection. In cases of multiple or no T2Hl, the lesion with the most dysplastic features was selected, and if multiple lesions had the same number of dysplastic features, the largest was chosen. Notably, in three cases, a lesion other than the largest was resected, documenting the influence of additional clinical factors on surgical decision-making. While this was not a prospective selection criterion, these observations may serve as a useful consideration for resection planning in similar cases.

Eltze *et al*. previously demonstrated that dysplastic lesions are better exposed on early unmyelinated MRIs.^16^ We confirmed this observation as we demonstrated that T2HI lesions in TSC on unmyelinated brain MRI harbour other dysplastic features, which may be already detectable at early MRI or become visible only after myelination. In addition, our results support our previous studies with the observation that a combination of highly dysplastic features is helpful in identifying the EL in TSC patients.^11,13^ The higher predictive value of a highly dysplastic lesion in the study of Hulshof *et al*. could be explained by their relatively older study population, as median age at surgery was 9.3 years in contrast to 23.0 months in the present study population, indicating a more severe phenotype in the present study population with often a more multi-focal epileptogenic zone. Nijman *et al*. suggested that the utility of structural MRI features, such as transmantle sign, cortical thickness, largest affected area and calcification, is limited in the identification of the EL in young children with TSC.^20^ Our findings confirm that the diagnostic accuracy of the combination of dysplastic features is much lower in young children, but show that in this age group T2-hypointensity is a better predictive marker for early identification of the EL in the process of pre-surgical evaluation of children with TSC and drug resistant epilepsy.

Although the histopathological similarities between FCD II and tubers in TSC are well-documented,^21^ their relation to age, level of myelination, and dysplastic features as observed in both histological examination and pre-surgical MRI has not been extensively studied. Some recent studies, such as studies using diffusion tensor imaging (DTI), have shown that parameters like fractional anisotropy (FA) and mean diffusivity (MD) can differentiate FCD from normal brain parenchyma^22^ others use techniques like neurite orientation imaging (NODDI)^23^ to highlight microstructural changes, such as myelin loss and abnormal myelin sheath formation. While this is an interesting area for future research, it exceeds the scope of this paper and could be the subject of subsequent studies.

There are several factors that may have contributed to an underestimation of the diagnostic value of T2-hypointensity in our study population. We evaluated a cohort of ‘severe’ TSC patients, as indicated by the high prevalence of *TSC2* mutations and young age both at seizure onset and at surgery. These patients are more likely to have a severe phenotype with high lesion burden,^24^ complicating the identification of the true EL with presence of multiple suspicious lesions and multi-focal epileptiform EEG activity. In this group pre-surgical evaluation methods are limited due to lack of cooperation or infeasibility of invasive recordings. Furthermore, there are two possible situations where T2-hypointensity might have correctly localized the EL, while resection did not result in post-operative seizure freedom. The majority of our study cohort presented with multiple T2HI lesions, in several cases not all inside the resected area. We found out that surgical failure was caused by the relapse from another EL in 33.3% of cases, possibly implicating that the discriminative value of T2-hypointensity was underestimated as this subgroup of patients maintained or acquired multiple true ELs. In addition, in 60% of cases surgical failure was supposedly caused by a larger extent of the EL, again indicating that the EL could have been identified correctly but was not resected completely. Both situations underline that seizure freedom is determined by both the predictive value of T2-hypointensity as well as complete resection of the supposed epileptogenic zone(s). Our pragmatic approach did not allow us to correct for these specific situations.

To our knowledge, this is the first study exploring the diagnostic value of T2-hypointensity in determining the EL in infants with TSC. Another strength of this study is the homogenous study cohort. Moreover, all MRIs were systematically evaluated by a trained team of experts, with substantial inter-observer agreement.^13^

The most important study limitation is the selected cohort consisting of TSC patients with a severe phenotype who are likely to be subjected to brain imaging at earlier age and are likely to have a higher lesion load. This however did not result in selection bias as it merely results in an underestimation of the discriminative power of T2-hypointensity. Secondly, the retrospective study design implicates variations in MRI protocols and timing of the first MRI. In addition, surgical strategy could differ between the two epilepsy centres. Finally, sample size of this study is relatively small. Surgical planning remains a process of combining different modalities to precisely identify the EL. Identification of T2HI lesions on unmyelinated brain MRI serves as an important radiological modality in the pre-surgical work-up of children with TSC and drug-resistant epilepsy and early MRI should therefore always be included in pre-surgical evaluation, if available. Predictive value of early unmyelinated brain MRI seems to surpass that of myelinated MRIs and early identification of T2HI lesions may improve surgical decision-making and allow for timely identification and resection of the EL in young children with TSC. The combinative value of T2-hypointensity and highly dysplastic lesions in defining the EL should be studied in a larger, less selected, and ideally prospective cohort.

## Conclusion

Assessing T2HI lesions on unmyelinated brain MRI has important diagnostic value in identifying the EL in pre-surgical work-up in infants with TSC and drug-resistant epilepsy. T2-hypointensity on unmyelinated brain MRI exceeds the diagnostic accuracy of a lesion being highly dysplastic.

## Data Availability

Data available on request due to privacy/ethical restrictions.
